# Diagnostic accuracy of real‑time point-of-care tracheal ultrasonography for the confirmation of proper endotracheal tube placement in neonatal acute care settings: a systematic review and diagnostic test accuracy meta-analysis

**DOI:** 10.1038/s41372-025-02461-4

**Published:** 2025-11-19

**Authors:** Mohammed Alsabri, Eslam Abady, Mohammed Tarek Hasan, Shree Rath, Ahmed Bostamy Elsnhory, Khaled Abouelmagd, Abdelrahman M. Tawfik, Ibrahim Qattea, Aysha Hasan

**Affiliations:** 1 Pediatric Emergency Department, Department of Pediatrics, St. Christopher’s Hospital for Children, Philadelphia, PA USA; 2 Faculty of Medicine, Tanta University, Tanta, Egypt; 3 Faculty of Medicine, Al-Azhar University, Cairo, Egypt; 4 All India Institute of Medical Sciences (AIIMS), Bhubaneswar, India; 5 Cardiology Department, Faculty of Medicine, Al-Azhar University, New Damietta, Egypt; 6 Fifth Year Medical Student, Smouha, Alexandria, Egypt; 7 Nassau University Medical Center, East Meadow, NY USA; 8 Anesthesiology Department, St. Christopher’s Hospital for Children, Philadelphia, PA USA

**Keywords:** Medical imaging, Diagnosis

## Abstract

Accurate confirmation of endotracheal tube (ETT) placement is critical in neonatal resuscitation. This systematic review and meta-analysis assessed the diagnostic accuracy of point-of-care ultrasound (POCUS) for confirming ETT placement in neonates. We searched PubMed, Scopus, Web of Science, and Cochrane Library through May 2025. Eligible studies included neonates (<28 days) and compared POCUS with gold-standard confirmation (capnography, chest radiography, or direct laryngoscopy). Diagnostic performance was pooled using a bivariate random-effects model. Thirteen studies (930 neonates) met inclusion criteria. POCUS showed pooled sensitivity of 93% and specificity of 59%, with an area under the SROC curve of 92%. tracheal placement was confirmed in 99%, and esophageal misplacement detected in 4%. Subgroup analysis indicated higher accuracy by neonatologists and with linear transducers. POCUS offers a rapid, radiation-free method for confirming neonatal ETT placement. Broader implementation requires standardized techniques and operator training.

## Introduction

Endotracheal intubation (ETI) is a critical life-saving procedure frequently performed in neonatal intensive care units (NICUs), delivery rooms, and during neonatal resuscitation to secure the airway and facilitate effective ventilation [1]. Despite its widespread application in airway management, the accurate and timely confirmation of proper endotracheal tube (ETT) placement remains a significant clinical challenge [2]. Misplacement of the ETT, particularly esophageal intubation, is a potentially catastrophic complication associated with severe morbidity and mortality, including hypoxia, cardiac arrest, brain injury, and even death [3, 4]. Therefore, reliable and rapid methods are needed for confirming ETT position.

Traditional methods of ETT confirmation in neonates include observation of chest movement, auscultation, and assessment of end-tidal carbon dioxide (ETCO₂). However, these methods are known to be unreliable, especially in noisy environments or in patients with underlying lung disease [5, 6]. The gold standard for confirming ETT placement has historically been capnography, which detects exhaled carbon dioxide. However, capnography can be misleading in conditions of absent or extremely low pulmonary blood flow where CO2 delivery to the lungs is minimal. Furthermore, capnography may be insufficient in the neonatal setting during resuscitation of extremely preterm infants or in low-perfusion states where CO2 delivery to the lungs is minimal [7].

In recent years, point-of-care ultrasonography (POCUS) has emerged as a valuable diagnostic modality across various disciplines, offering real-time, non-invasive assessment bedside [8]. Tracheal ultrasonography has garnered increasing attention as a promising technique for confirming ETT placement. This technique involves visualizing the trachea and esophagus, allowing for the direct identification of the ETT within the tracheal lumen or its aberrant placement in the esophagus [9, 10]. Tracheal ultrasonography offers important advantages such as its non-invasive nature, rapid execution, and applicability in situations where capnography may be unavailable.

Several studies have evaluated the diagnostic accuracy of tracheal ultrasonography for confirming ETT placement in neonates [11, 12]. However, their findings vary widely due to differences in technique, operator expertise, gestational age groups, and reference standards used. Previous systematic reviews have predominantly focused on adult or mixed populations, leaving a knowledge gap specific to neonates in acute settings. This highlighted the necessity for a updated comprehensive synthesis of the existing evidence.

Therefore, the objective of this systematic review and meta-analysis is to comprehensively evaluate the diagnostic accuracy of real-time point-of-care tracheal ultrasonography in confirming endotracheal tube placement in neonatal patients.

## Methods

This systematic review and meta-analysis was registered with the international Prospective Register of Systematic Reviews (PROSPERO) database with registration number (CRD420251082454). We reported this study according to the Preferred Reporting Items for Systematic Reviews and Meta-analysis (PRISMA) statement guidelines [13].

### Search strategy

To identify relevant studies, we performed comprehensive electronic searches across PubMed, Web of Science, Scopus, and the Cochrane Library databases, covering all available records from inception through May 2025. Our search strategy incorporated both Medical Subject Headings (MeSH) terms and keyword variations related to “endotracheal intubation”, “ultrasonography”, acute care settings (like “emergency” and “ICU”), and diagnostic accuracy combined with Boolean operators. All details on the search methodology and the full search strategy for each database are provided in Supplementary Table. 1. Additionally, we scanned the reference lists of the included articles to identify any further eligible studies.

### Eligibility criteria

Eligibility criteria were strictly defined to maintain relevance of studies. We selected observational or interventional studies that reported diagnostic accuracy data—specifically, studies providing true positives, false positives, true negatives, and false negatives, either directly or in a format allowing these values to be derived. Eligible studies had to evaluate ultrasonography for ETT placement confirmation in neonates (under 28 days old) within ED or ICU settings. The accuracy of ultrasonography required verification by an accepted reference standard such as capnography or direct visualization via laryngoscopy visualizing the ETT) passing through the vocal cords/glottis during or post-intubation when clinical response was inadequate.

Exclusion criteria encompassed case reports, cadaveric or animal studies, studies reporting on pediatric subjects, and conference abstracts lacking full-text data, and studies not in English Language.

### Screening

Two independent blinded reviewers assessed the studies according to the eligibility criteria. Conflicts were resolved through discussion or by consulting a third independent reviewer. After removing duplicated of the retrieved references, the titles and abstracts of the articles were screened by the reviewers. We then screened full-text articles of the eligible abstracts for final inclusion in the systematic review.

### Data extraction and outcomes

The following data were extracted by two independent authors into a standardized spreadsheet, for each eligible study: (1) *Study information:* country, study design, setting (ER/ICU), sample size, period of recruitment, ultrasound technique, operator type, probe type, timing of US, type of reference standard, main inclusion criteria, primary endpoints, and the conclusion of the study; (2) *Patient baseline characteristics:* age, sex, birth weight, indication for intubation, medical history, admission timing (daytime/night), intubation difficulty (Cormack and Lehane grade 3 or 4), and intubation time, (3) POCUS *techniques and ultrasonographic signs:* the primary POCUS technique reported across the included studies involved a transverse view of the neck at the level of the suprasternal notch. Correct tracheal intubation was typically confirmed by visualizing a single hyperechoic interface with posterior shadowing, representing the ETT within the trachea. In contrast, esophageal intubation was often identified by a ‘double-tract’ or ‘second target’ sign, where two tube-like structures (the trachea and the esophagus containing the ETT) were visualized; (4) *Outcome measures:* Endotracheal intubation (events), esophageal intubation (events), sensitivity, specificity, Area under ROC Curve (AUC), true positive, true negative, false positive, false negative, diagnostic odds ratio, predictive value (positive, negative), likelihood ratio (positive, negative), time to ETT confirmation by POCUS, time to ETT confirmation by reference standard, missed esophageal intubation rate, reintubation due to misplacement, POCUS-related complications, undetected esophageal intubation by POCUS, time to adequate view. Data extraction was conducted systematically, with one reviewer collecting study details and another verifying accuracy.

### Quality assessment

Study quality was appraised using the QUADAS-2 tool, which evaluates risk of bias across four domains: patient selection, index test, reference standard, and flow/timing. Two investigators independently conducted assessments, with disagreements resolved by consensus [14].

### Statistical analysis

The statistical analysis for this meta-analysis was conducted using R software (version 4.3.0) with the “meta” and “metafor” packages. Diagnostic accuracy measures, including sensitivity and specificity, were pooled using a bivariate random-effects model to account for between-study heterogeneity and the intrinsic correlation between sensitivity and specificity. Summary receiver operating characteristic (SROC) curves were generated to evaluate the overall diagnostic performance of POCUS for ETT confirmation, with the area under the curve (AUC) serving as a measure of discriminative ability.

For dichotomous variables, such as incidence of esophageal intubation, the proportions and totals were pooled as odds ratios (OR). For continuous outcomes, such as time to ETT confirmation, mean differences (MD) with 95% confidence intervals (CI) were calculated using inverse-variance random-effects models. Heterogeneity was assessed using the I² statistic and Cochran’s Q test, with I² > 50% or a *p*-value < 0.10 indicating substantial heterogeneity.

Publication bias was evaluated using funnel plots and Egger’s regression test for asymmetry, with *p* < 0.05 suggesting potential bias. Sensitivity analyses were conducted by sequentially excluding individual studies to assess the robustness of pooled estimates. All statistical tests were two-tailed, with *p* < 0.05 considered statistically significant.

## Results

### Screening and study selection

**Fig. 1:**
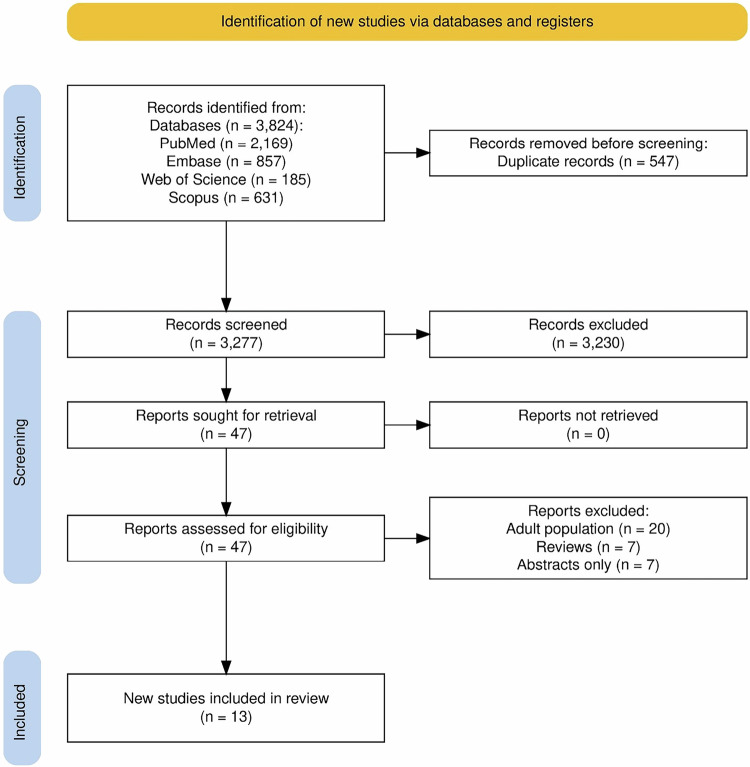
PRISMA flowchart of screening and study selection.

A total of 3824 records were obtained on initial search, of which 3277 unique records were further assessed for inclusion based on a title and abstract screening. Of these, the full texts of 47 records were assessed for inclusion. Finally, 13 studies were included for qualitative and quantitative synthesis (Fig. 1). The list and characteristics of all included studies are provided in Supplementary Table 2 [refs. 15–27]

### Baseline and study characteristics

Of the 13 included studies with 930 neonates, four were cross-sectional, one was retrospective and the remaining were prospective cohort studies. All studies included neonates admitted to the NICU. Operators of POCUS were either neonatologists, nurses or radiologists. Weighted average mean of gestational age 32.07 ± 5.10 weeks, while neonate’s chronological age at Intubation range form 2 to 16 days. Majority of the included neonates were male. Further details are presented in Supplementary Table 2.

### Quality assessment

Quality assessment using QUADAS-2 revealed Low to Some concerns across all studies except Takeuchi and de Kock et al., which demonstrated High risk of bias due to confounding in reference standard and patient selection. Across applicability domains, most studies demonstrated some concerns to low risk of bias except Takeuchi et al., which demonstrated high risk of bias due to confounding in reference standard. These studies were retained in the primary meta-analysis to ensure comprehensiveness, and their influence on the pooled estimates was evaluated through sensitivity analyses. Further details are provided in Supplementary Figs. 1, 2.

### Outcomes

#### *POCUS-confirmed success rate* “POCUS diagnostic accuracy for correctly placed ETTs”

**Fig. 2:**
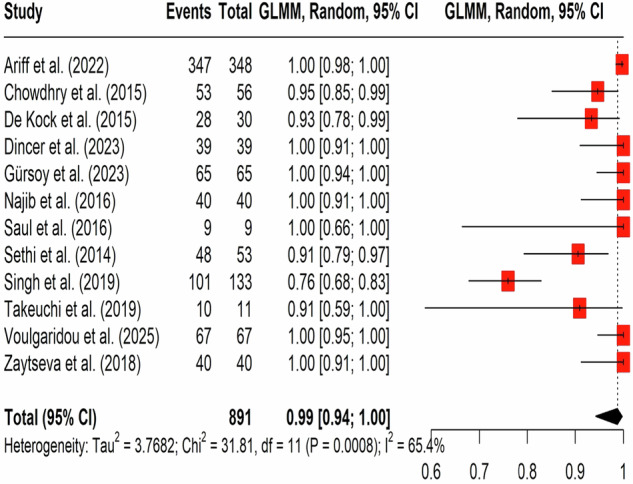
Forest plot of POCUS-confirmed success rate.

Success rate was defined as the proportion of neonates with correctly placed ETTs confirmed by POCUS as confirmed by reference standards (not the procedural success of intubation itself), form pooled analysis of 12 studies with 891 patients revealed a 99% odds of success of ET tube insertion when confirmed by POCUS (GLMM: 0.99; 95% CI: 0.94, 1.0) (Fig. 2) The assessment demonstrated high heterogeneity (I2 = 65.4%, *P* = 0.0008), which resolved on sensitivity analysis by omitting Singh et al. (I2 = 8%), with no change in effect size (0.99(95% CI: 0.96, 1)) (Supplementary Fig. 3). Funnel plot by Egger’s test revealed significant publication bias (*p* < 0.0001) (Supplementary Fig. 4).

#### Diagnostic test accuracy of POCUS for ETT confirmation

**Fig. 3:**
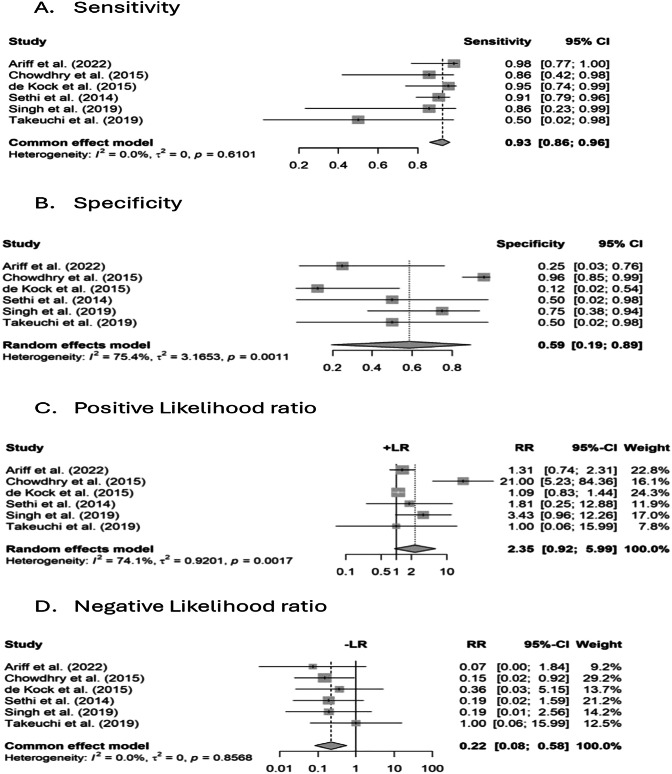
Meta-analysis of diagnostic accuracy of POCUS for confirmation. **A** Sensitivity of POCUS across included studies with pooled estimate and heterogeneity statistics. **B** Specificity of POCUS with pooled estimate and heterogeneity statistics. **C** Positive likelihood ratio (+LR) with pooled estimate. **D** Negative likelihood ratio (–LR) with pooled estimate. Squares represent study-specific estimates, horizontal lines indicate 95% CIs, and diamonds show pooled effects.

**Fig. 4:**
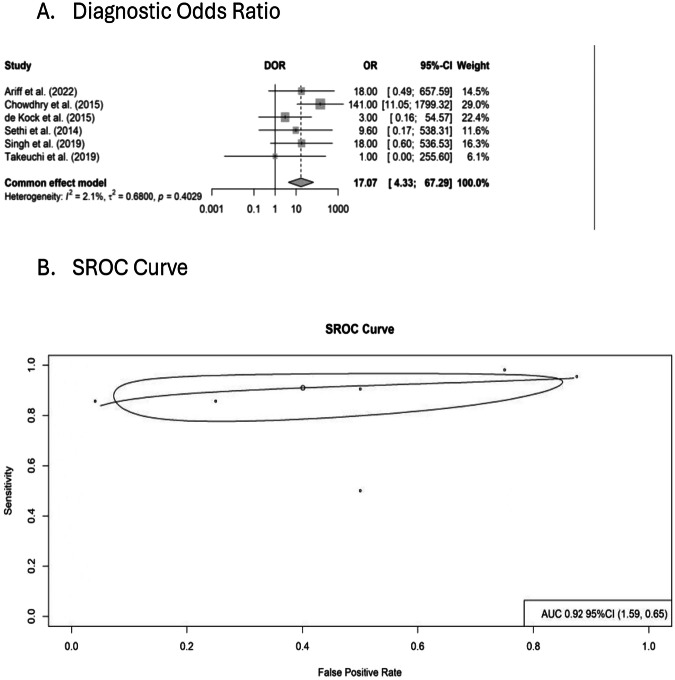
Diagnostic odds ratio and summary receiver operating characteristic (SROC) curve of POCUS for confirmation of endotracheal tube (ETT) placement. **A** Forest plot of diagnostic odds ratios (DOR) for individual studies with pooled estimate shown as a diamond. **B** SROC curve plotting sensitivity against false positive rate, with area under the curve (AUC) and confidence region displayed. Circles represent individual study estimates.

Pooled analysis of five studies revealed a high sensitivity of 93%(95% CI: 0.86, 0.96) and a moderate specificity of 59% (95% CI: 0.19, 0.89) (Fig. 3A, B). A positive likelihood ratio of 2.35 (95% CI: 0.92, 5.99) and a negative likelihood ratio of 0.22 (95% CI: 0.08, 0.58) was obtained (Fig. 3C, D). Further, pooled estimate demonstrated a diagnostic odds ratio of 17.07 (95% CI: 4.33, 67.29) (Fig. 4A). SROC curve revealed an AUC of 92% (95% CI: 1.59, 0.65) (Fig. 4B).

#### Esophageal intubation

**Fig. 5:**
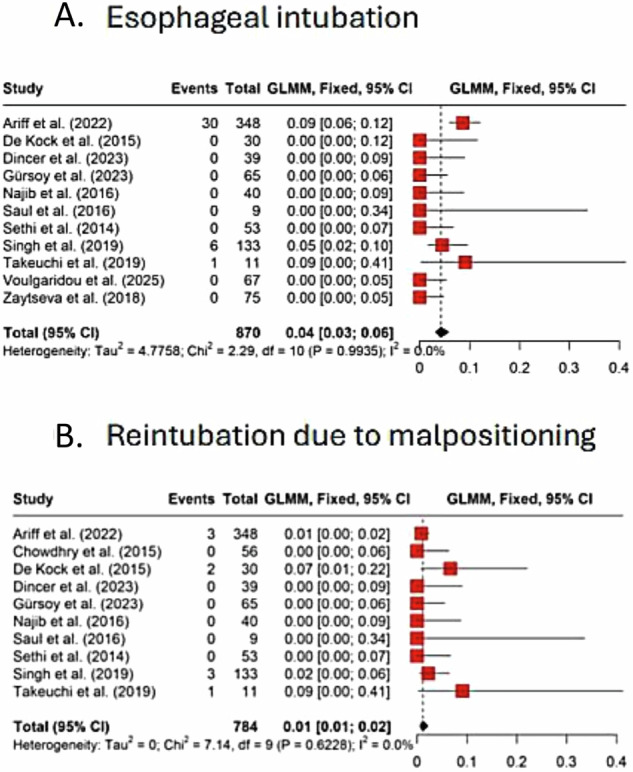
Incidence of esophageal intubation and reintubation due to malpositioning. **A** Forest plot of esophageal intubation events across studies, with pooled incidence shown as a diamond. **B** Forest plot of reintubation due to malpositioning, with pooled incidence shown as a diamond. Squares represent study-specific estimates, horizontal lines indicate 95% CIs, and diamonds show pooled effects.

The pooled analysis of 11 studies with 870 neonates recorded an incidence of esophageal intubation across the studies was 4% of total attempts (GLMM: 0.04; 95% CI: 0.03, 0.06) (Fig. 5A), with homogenous estimate (I2 = 0%, *P* = 0.99). Funnel plot by Egger’s test revealed significant publication bias (*p* < 0.0001) (Supplementary Fig. 5).

#### Reintubation due to misplacement

Pooled analysis of 10 studies revealed that reintubation was required in 1% proportion of included neonates due to initial tube misplacement (GLMM: 0.01; 95% CI: 0.01, 0.02) (Fig. 5B), with homogenous estimate (I2 = 0%, *P* = 0.623). Funnel plot by Egger’s test revealed no publication bias (*p* = 0.9) (Supplementary Fig. 6).

## Discussion

Accurate and timely confirmation of ETT placement in neonates is a critical determinant of successful airway management in acute care settings, including NICUs and delivery rooms. Our systematic review and diagnostic test accuracy (DTA) meta-analysis demonstrated that POCUS is a highly sensitive (93%) and moderately specific (59%) modality for confirming correct ETT placement in neonates, with strong overall diagnostic performance (AUC = 92%), highlighting its diagnostic potential in real-world acute care settings. The overall proportion of neonates with correctly placed ETTs successfully confirmed by POCUS as verified against reference standards tests was 99%, while the proportion of esophageal intubations was 4%, and only 1% of neonates required reintubation due to initial misplacement of the ETT. “ In our analysis, a ‘positive test’ indicates the sonographic identification of the ETT within the tracheal lumen, while a ‘negative test’ indicates its absence from the trachea, suggesting esophageal placement with diagnostic accuracy assessed against established reference standards”.

The pooled sensitivity of 93% is consistent with previous meta-analyses conducted across broader populations, which reported sensitivity ranging from 91 to 98% for POCUS in detecting correct ETT positioning [10, 28]. However, our analysis offers critical value by focusing exclusively on neonatal populations, thereby addressing an existing evidence gap. The relatively moderate specificity (59%) observed may reflect a higher false-positive rate, possibly attributable to air artifacts or esophageal movement being misinterpreted as tracheal placement, especially by less experienced operators [29]. Nonetheless, the high sensitivity and a negative likelihood ratio (NLR) of 0.22 suggest POCUS is particularly valuable as a rule-out test in emergency settings where rapid clinical decisions are paramount.

Our diagnostic odds ratio (DOR) of 17.07 is noteworthy, indicating strong discriminatory power and clinical utility. The findings support the notion that tracheal ultrasonography offers a reliable alternative to capnography, especially in circumstances where traditional methods may be limited—such as poor perfusion states, ongoing chest compressions, or in extremely low birth weight neonates [30, 31].

POCUS presents a unique combination of advantages for neonatal care: it is non-invasive, radiation-free, repeatable, and provides real-time feedback. Its portability enables immediate bedside use during resuscitation, unlike chest radiography which introduces delays and logistical challenges [32]. Given its ability to visualize both tracheal and esophageal anatomy, POCUS also enables rapid identification of esophageal intubation—a potentially fatal event if missed. In our pooled analysis, the esophageal intubation rate was 4%, highlighting the relevance of a reliable verification tool. Importantly, the need for reintubation due to misplacement (e.g., incorrect depth) was only 1%, suggesting that early detection by POCUS can lead to prompt correction of malpositioned tubes, thereby improving outcomes. This demonstrates the utility of POCUS not just for verifying tracheal placement, but also for assessing tube depth.

Recent guidelines and consensus documents have also begun recognizing the potential role of airway ultrasound in neonatal and paediatric airway management. A 2022 expert consensus by the REPEM POCUS collaborators highlighted the growing utility of bedside ultrasound in neonatal intubation procedures, citing its ability to reduce confirmation time and misplacement risk [33]. While the diagnostic metrics are encouraging, POCUS remains inherently operator-dependent. Studies included in our analysis used varied operator profiles, including neonatologists, radiologists, and trained nurses. As noted in recent evaluations by Al-Absi et al., structured training programs significantly enhance the diagnostic yield of POCUS in imaging and diagnostics [34]. Future consideration of incorporating POCUS training into neonatal resuscitation protocols, such as the Neonatal Resuscitation Program (NRP), may improve clinician proficiency and standardize usage.

Despite robust findings, heterogeneity was moderate to high in some pooled estimates. This may reflect differences in ultrasound techniques (trans-thyroidal vs. suprasternal views), variability in reference standards (capnography vs. direct laryngoscopy), and gestational age groups. For example, extremely premature neonates pose technical challenges due to smaller airway diameters and reduced anatomical landmarks. Our sensitivity analysis showed that heterogeneity decreased significantly upon exclusion of outlier studies such as Singh et al., suggesting variability in methodology or patient selection as sources of inconsistency.

Additionally, although the AUC was high, the wide confidence interval around specificity and the presence of publication bias—particularly for success and esophageal intubation rates—indicate potential selective reporting. Many studies with negative or neutral findings may not have been published. Moreover, the majority of studies had some concern in quality assessment domains, with two studies (Takeuchi and de Kock et al.) showing high risk of bias due to inadequate reference standards and selection issues. A further limitation is the inability to perform robust subgroup analyses based on operator experience, ultrasound technique, or equipment type due to the limited number of studies and inconsistent reporting of these variables. Future research with larger, more detailed studies will be needed to explore how these factors influence the diagnostic accuracy of POCUS. Future studies should employ standardized diagnostic frameworks and ensure blinding to reduce bias.

Our findings advocate for the broader integration of tracheal POCUS into neonatal intubation algorithms, especially in circumstances where capnography may be unreliable, such as in neonates with poor perfusion states or during chest compressions. Future research should prioritize RCTs comparing POCUS-guided confirmation versus traditional methods in emergency and elective neonatal intubations. Standardization of training protocols is essential, allowing for routine and evidence-guided management of neonates.

## Conclusion

Real-time point-of-care tracheal ultrasonography is a highly sensitive and moderately specific tool for confirming the correct site of endotracheal tube placement in neonates in acute care settings. It offers rapid, bedside, radiation-free confirmation and may reduce the incidence of undetected esophageal intubation. While variability in technique and operator skill remains a limitation, the overall diagnostic accuracy and safety profile of POCUS support its increasing adoption in neonatal airway management protocols. Despite methodological heterogeneity across studies, this analysis provides the most comprehensive evidence to date supporting POCUS as a valuable adjunctive tool for neonatal endotracheal tube placement verification, while highlighting the need for standardized protocols and training programs. Larger, high-quality prospective studies and implementation trials are warranted to further consolidate its role and optimize training frameworks.

## Supplementary information

Supplementary Information File

## Data Availability

All data and materials will be available upon official request from the authors.

## References

[1] Wyllie JP. Neonatal endotracheal intubation. Arch Dis Child Educ Pr Ed. 2008;93:44–49. 10.1136/adc.2007.121160. 10.1136/adc.2007.121160 18356305

[2] Rudraraju P, Eisen LA. Confirmation of endotracheal tube position: a narrative review. J Intensive Care Med. 2009;24:283–92. 10.1177/0885066609340501. 19654121 10.1177/0885066609340501

[3] Katz SH, Falk JL. Misplaced endotracheal tubes by paramedics in an urban emergency medical services system. Ann Emerg Med. 2001;37:32–37. 10.1067/mem.2001.112098. 11145768 10.1067/mem.2001.112098

[4] Miller KA, Kimia A, Monuteaux MC, Nagler J. Factors associated with misplaced endotracheal tubes during intubation in pediatric patients. J Emerg Med. 2016;51:9–18. 10.1016/j.jemermed.2016.04.007. 27236246 10.1016/j.jemermed.2016.04.007

[5] Schmölzer GM, Roehr CC. Techniques to ascertain correct endotracheal tube placement in neonates. Cochrane Database Syst Rev. 2014;9:CD010221. 10.1002/14651858.CD010221.pub2. 10.1002/14651858.CD010221.pub2 25217732

[6] Schmölzer GM, O’Reilly M, Davis PG, Cheung PY, Roehr CC. Confirmation of correct tracheal tube placement in newborn infants. Resuscitation. 2013;84:731–7. 10.1016/j.resuscitation.2012.11.028. 23211476 10.1016/j.resuscitation.2012.11.028

[7] Williams E, Dassios T, Greenough A. Carbon dioxide monitoring in the newborn infant. Pediatr Pulmonol. 2021;56:3148–56. 10.1002/ppul.25605. 34365738 10.1002/ppul.25605

[8] Maw AM, Huebschmann AG, Mould-Millman NK, Dempsey AF, Soni NJ. Point-of-care ultrasound and modernization of the bedside assessment. J Grad Med Educ. 2020;12:661–5. 10.4300/JGME-D-20-00216.1. 33391586 10.4300/JGME-D-20-00216.1 PMC7771602

[9] Karacabey S, Sanrı E, Gencer EG, Guneysel O. Tracheal ultrasonography and ultrasonographic lung sliding for confirming endotracheal tube placement: speed and reliability. Am J Emerg Med. 2016;34:953–6. 10.1016/j.ajem.2016.01.027. 26994679 10.1016/j.ajem.2016.01.027

[10] Gottlieb M, Holladay D, Peksa GD. Ultrasonography for the confirmation of endotracheal tube intubation: a systematic review and meta-analysis. Ann Emerg Med. 2018;72:627–36. 10.1016/j.annemergmed.2018.06.024. 30119943 10.1016/j.annemergmed.2018.06.024

[11] Dennington D, Vali P, Finer NN, Kim JH. Ultrasound confirmation of endotracheal tube position in neonates. Neonatology. 2012;102:185–9. 10.1159/000338585. 22777009 10.1159/000338585

[12] Descamps CS, Beissel A, Vo Van P, Butin M, Stacoffe N, Giai J, et al. Role of ultrasonography in the assessment of correct endotracheal tube placement in neonates. Acta Paediatr. 2020;109:1057–9. 10.1111/apa.15097. 31737938 10.1111/apa.15097

[13] Page MJ, McKenzie JE, Bossuyt PM, Boutron I, Hoffmann TC, Mulrow CD, et al. The PRISMA 2020 statement: an updated guideline for reporting systematic reviews. BMJ. 2021;372:n71 10.1136/bmj.n71. 33782057 10.1136/bmj.n71 PMC8005924

[14] Whiting PF, Rutjes AW, Westwood ME, Mallett S, Deeks JJ, Reitsma JB, et al. QUADAS-2: a revised tool for the quality assessment of diagnostic accuracy studies. Ann Intern Med. 2011;155:529–36. 10.7326/0003-4819-155-8-201110180-00009. 22007046 10.7326/0003-4819-155-8-201110180-00009

[15] Ariff S, Ali KQ, Tessaro MO, Ansari U, Morris S, Soofi SB, et al. Diagnostic accuracy of point-of-care ultrasound compared to standard-of-care methods for endotracheal tube placement in neonates. Pediatr Pulmonol. 2022;57:1744–50. 10.1002/ppul.25955. 35501297 10.1002/ppul.25955

[16] Chowdhry R, Dangman B, Pinheiro JMB. The concordance of ultrasound technique versus X-ray to confirm endotracheal tube position in neonates. J Perinatol. 2015;35:481–4. 10.1038/jp.2014.240. 25611791 10.1038/jp.2014.240

[17] De Kock SH, Otto SF, Joubert G. The feasibility of determining the position of an endotracheal tube in neonates by using bedside ultrasonography compared with chest radiographs. S Afr J Child Health. 2015;9:3. 10.7196/sajch.740.

[18] Dennington D, Vali P, Finer NN, Kim JH. Ultrasound confirmation of endotracheal tube position in neonates: prospective pilot study. Neonatology. 2012;102:185–9. 10.1159/000338585. 22777009 10.1159/000338585

[19] Dincer E, Topçuoğlu S, Karatekin G. Ultrasonography for determining endotracheal tube tip position in very low birth weight infants. J Ultrasound Med. 2023;42:437–41. 10.1002/jum.16067. 35904138 10.1002/jum.16067

[20] Gürsoy BK, Dilli D, Sarıkaya Y, Akduman H, Çitli R, Örün UA, et al. Ultrasonographic evaluation of endotracheal tube position in newborns with CHD. Cardiol Young-. 2023;33:2049–53. 10.1017/S1047951122003420. 36517980 10.1017/S1047951122003420

[21] Najib K, Pishva N, Amoozegar H, Pishdad P, Fallahzadeh E. Ultrasonographic confirmation of endotracheal tube position in neonates. Indian Pediatr. 2016;53:886–8. 10.1007/s13312-016-0953-6. 27771669 10.1007/s13312-016-0953-6

[22] Saul D, Ajayi S, Schutzman DL, Horrow MM. Sonography for complete evaluation of neonatal intensive care unit central support devices: a pilot study. J Ultrasound Med. 2016;35:1465–73. 10.7863/ultra.15.06104. 27229130 10.7863/ultra.15.06104

[23] Sheth M, Jaeel P, Nguyen J. Ultrasonography for verification of endotracheal tube position in neonates and infants. Am J Perinatol. 2016;34:627–32. 10.1055/s-0036-1597846. 28030872 10.1055/s-0036-1597846

[24] Singh P, Thakur A, Garg P, Aggarwal N, Kler N. Normative data of optimally placed endotracheal tube by point-of-care ultrasound in neonates. Indian Pediatr. 2019;56:374–80. 31102379

[25] Takeuchi S, Arai J, Nagafuji M, Hinata A, Kamakura T, Hoshino Y, et al. Ultrasonographic confirmation of endotracheal intubation in extremely low birthweight infants – secondary publication. Pediatr Int. 2020;62:175–9. 10.1111/ped.14069. 31785124 10.1111/ped.14069

[26] Voulgaridou A, Deftereos S, Chloropoulou P, Bekiaridou K, Tsouvala E, Meziridou R, et al. Emergency identification of endotracheal tube tip via ultrasonography used by trained nurse in the neonatal intensive care unit. Diagnostics. 2025;15:262. 10.3390/diagnostics15030262. 39941192 10.3390/diagnostics15030262 PMC11817360

[27] Zaytseva A, Kurepa D, Ahn S, Weinberger B. Determination of optimal endotracheal tube tip depth from the gum in neonates by X-ray and ultrasound. J Matern Fetal Neonatal Med. 2020;33:2075–80. 10.1080/14767058.2018.1538350. 30332898 10.1080/14767058.2018.1538350

[28] Li X, Zhang J, Karunakaran M, Hariharan VS. Diagnostic accuracy of ultrasonography for the confirmation of endotracheal tube intubation: a systematic review and meta-analysis. Med Ultrason. 2023;25:72–81. 10.11152/mu-3594. 36780595 10.11152/mu-3594

[29] Lau YH, See KC. Point-of-care ultrasound for critically ill patients: a mini-review of key diagnostic features and protocols. World J Crit Care Med. 2022;11:70–84. 10.5492/wjccm.v11.i2.70. 35433316 10.5492/wjccm.v11.i2.70 PMC8968483

[30] Williams EE, Dassios T, Harris C, Greenough A. Capnography waveforms: basic interpretation in neonatal intensive care. Front Pediatr. 2024;12:1396846. 10.3389/fped.2024.1396846. 38638588 10.3389/fped.2024.1396846 PMC11024230

[31] Williams E, Dassios T, O’Reilly N, Walsh A, Greenough A. End-tidal capnography monitoring in infants ventilated on the neonatal intensive care unit. J Perinatol. 2021;41:1718–24. 10.1038/s41372-021-00978-y. 33649438 10.1038/s41372-021-00978-y PMC7917950

[32] Lanspa MJ, Fox SW, Sohn J, Dugar S, Klick JC, Diaz-Gomez J, et al. Definitive advantages of point-of-care ultrasound: a case series. CASE. 2022;6:293–8. 10.1016/j.case.2022.05.008. 36036052 10.1016/j.case.2022.05.008 PMC9399626

[33] Parri N, Berant R, Giacalone M, Jones SD, Friedman N, REPEM POCUS collaboration. Dissemination and use of point-of-care ultrasound by pediatricians in Europe: a research in European pediatric emergency medicine network collaborative survey. Pediatr Emerg Care. 2022;38:e1594–e600. 10.1097/PEC.0000000000002767. 35608533 10.1097/PEC.0000000000002767

[34] Al-Absi DT, Simsekler MCE, Omar MA, Soliman-Aboumarie H, Abou Khater N, Mehmood T, et al. Evaluation of point-of-care ultrasound training among healthcare providers: a pilot study. Ultrasound J. 2024;16:12. 10.1186/s13089-023-00350-5. 38383673 10.1186/s13089-023-00350-5 PMC10881927

