# Supplementary material for: Diagnostic accuracy of real‑time point-of-care tracheal ultrasonography for the confirmation of proper endotracheal tube placement in neonatal acute care settings: a systematic review and diagnostic test accuracy meta-analysis
Source: J Perinatol. 2025 Nov 19;46(1):12–8. doi: 10.1038/s41372-025-02461-4 (PMC12815668; doi:10.1038/s41372-025-02461-4)

Supplementary Files

Supplementary Table 1: Search strategy across databases

Database	Search Strategy
**PubMed**	(("Ultrasonography"[MeSH Terms] OR "Point-of-Care Systems"[MeSH Terms] OR ultrasonography OR "point-of-care ultrasound" OR POCUS OR "bedside ultrasound" OR sonography) AND ("Intubation, Intratracheal"[MeSH Terms] OR intubation OR "endotracheal intubation" OR "airway management" OR "tracheal intubation" OR "ETT placement" OR "endotracheal tube" OR "tube placement") AND ("Diagnosis"[MeSH Terms] OR "Diagnostic Techniques and Procedures"[MeSH Terms] OR diagnosis OR diagnostic OR diagnostics OR "diagnostic accuracy" OR "diagnostic tool" OR "diagnostic imaging") AND (ICU OR "intensive care unit" OR "critical care" OR "critical care unit" OR "intensive therapy unit" OR ITU OR ER OR "emergency room" OR "emergency department" OR "emergency ward" OR "emergency service" OR "emergency medicine"))
**Scopus**	TITLE-ABS-KEY(ultrasonography OR "point-of-care ultrasound" OR POCUS OR "bedside ultrasound" OR sonography) AND TITLE-ABS-KEY(intubation OR "endotracheal intubation" OR "airway management" OR "tracheal intubation" OR "ETT placement" OR "endotracheal tube" OR "tube placement") AND TITLE-ABS-KEY(diagnosis OR diagnostic OR diagnostics OR "diagnostic accuracy" OR "diagnostic tool" OR "diagnostic imaging" OR "diagnostic procedure") AND TITLE-ABS-KEY(ICU OR "intensive care unit" OR "critical care" OR "critical care unit" OR "intensive therapy unit" OR ITU OR ER OR "emergency room" OR "emergency department" OR "emergency ward" OR "emergency service" OR "emergency medicine")
**Web of Science**	TS=(ultrasonography OR "point-of-care ultrasound" OR POCUS OR "bedside ultrasound" OR sonography) AND TS=(intubation OR "endotracheal intubation" OR "airway management" OR "tracheal intubation" OR "ETT placement" OR "endotracheal tube" OR "tube placement") AND TS=(diagnosis OR diagnostic OR diagnostics OR "diagnostic accuracy" OR "diagnostic tool" OR "diagnostic imaging" OR "diagnostic procedure") AND TS=(ICU OR "intensive care unit" OR "critical care" OR "critical care unit" OR "intensive therapy unit" OR ITU OR ER OR "emergency room" OR "emergency department" OR "emergency ward" OR "emergency service" OR "emergency medicine")
**Embase**	('ultrasonography'/exp OR 'point of care ultrasound':ti,ab OR ultrasonography:ti,ab OR pocus:ti,ab OR 'bedside ultrasound':ti,ab OR sonography:ti,ab) AND ('endotracheal intubation'/exp OR intubation:ti,ab OR 'endotracheal intubation':ti,ab OR 'airway management':ti,ab OR 'tracheal intubation':ti,ab OR 'ett placement':ti,ab OR 'endotracheal tube':ti,ab OR 'tube placement':ti,ab) AND ('diagnostic test'/exp OR diagnosis:ti,ab OR diagnostic:ti,ab OR 'diagnostic tool':ti,ab OR 'diagnostic accuracy':ti,ab) AND ('intensive care unit'/exp OR icu:ti,ab OR 'emergency room'/exp OR er:ti,ab) AND [humans]/lim NOT [conference abstract]/lim

Supplementary table 2: Summary and baseline characteristics of included population

Study	GA/Age at intubation	Country	Study design	Sample size	Main inclusion criteria	Exclusion criteria	Setting (ER/PICU/NICU)	Period of recruitment	Operator type	Operator Training/Experience for US	Ultrasound technique	Probe/Transducer Type	Probe/Transducer Frequency (MHz)	Specific POCUS Signs	Timing of US	Type of Reference Standard (Gold Standard)	Capnography used (yes/no)	Chest ultrasound used (yes/no)
Ariff et al. (2022)	32.6 ± 4.8 weeks GA / median of 2 days	Pakistan	Cross-sectional diagnostic accuracy	348	Neonates requiring intubation	Abnormal airway anatomy	NICU/LR/OR	Jul 2018-Jun 2019	Neonatologists, fellows, residents, nurses	Simulator-trained (OSATS certified)	Transtracheal/transthoracic	Linear (L12-4)	5-12 (estimated)	Double-tract (tracheal), Single-tract (esophageal)	Median 3 sec (IQR 3-4)	Auscultation + capnography + CXR	Yes	Yes (ETT only)
Chowdhry et al. (2015)	28.3 ± 4.7 weeks GA	USA	Prospective observational clinical trial	29 (56 image pairs)	NICU patients with ETT requiring X-ray	Congenital heart disease	50-bed regional NICU	Nov 2012 – Nov 2013	US technologists/pediatric radiologists (13)	1-hour training by pediatric radiologist	Parasternal approach; aortic arch apex to ETT tip	Phillips CX-50 curved	8–5 MHz	ETT tip <1 cm from aortic arch = deep	Within 1 hour of X-ray (mean: 38 ± 28 min)	X-ray (ETT tip vs. T3 vertebra)	No	Yes
de Kock 2015	median of 13.5 days	South Africa	Prospective cross-sectional	30	Intubated neonates in NICU	Active resuscitation, no consent	NICU	3 months	Radiology registrar	Radiology training	Aortic arch-ETT tip distance measurement	Curvilinear	6	ETT tip position relative to aortic arch	Median 48min post-CXR	CXR (T1-T2 as gold standard)	No	Yes
Dincer 2022	NR (GA: 26 weeks median)	Turkey	Prospective observational	24 VLBW infants	<34 wks, <1500g	Cardiac/pulm anomalies, poor visualization	NICU	Jul 2017-Apr 2018	Neonatologist	Experienced in echo/LUS	Midsagittal ETT-carina	Phased array	4-12Hz	RPA/carina	Immediate post-intub	CXR	No	Yes
Gürsoy 2022	37.8 ± 2.19 weeks GA	Turkey	Prospective observational	65	Intubated CHD neonates	Airway anomalies, poor XR, no consent	NICU	2020-21	Neonat	6mo+	ETT-carina	Linear	13MHz	RPA/carina	2h post-XR	CXR	No	Yes
Najib et al. (2016)	16±17 days	Iran	Cross-sectional	40	Neonates requiring intubation in NICU	ETT tip not visible (n=17), poor radiography quality (n=13)	NICU (3 centers)	May-Oct 2014 (6 months)	Neonatologist supervised by radiologist/cardiologist	6 months chest sonography training	Midsagittal views using RPA as carina surrogate	Linear array	10 MHz	ETT tip-to-carina distance	Within 1 hour of radiography	Chest X-ray (blinded)	No	Yes
Sethi et al. (2014)	36.1 ± 2.85 weeks / mean 3.89 days	India	Prospective observational	53	• NICU intubations • All gestational ages	• Delivery room intubations • Emergency room intubations	NICU	Aug 2011-Sep 2012	Single neonatologist	• 2-week radiologist training • Prior neonatal US experience	High parasternal view measuring ETT-aortic arch distance	Curvilinear (Sonosite MicroMaxx)	5-8 MHz	• Aortic arch identification • ETT tip movement with gentle manipulation	Post-intubation (mean 19.3 min)	Chest X-ray (T1-T3 vertebral level)	No	Yes
Saul et al 2016	31.0 ± 5.1/ 3.89 ± 5.45 days	USA	Prospective blinded diagnostic accuracy	25 (50 devices)	NICU patients with ETT/UVC/UAC/PICC placed/adjusted	Unstable patients, no consent	NICU	Not specified	Radiology resident + attending	Resident (4 yrs experience), Attending (30 yrs experience)	Anatomic survey + dedicated catheter views	Linear/curved array	5-17 MHz	Catheter tip location, thrombosis	Within 24h of radiography	Radiography	No	Yes
Singh et al. (2019)	30.8 ± 4.6 weeks PMA	India	Cross-sectional	133	• Neonates requiring intubation • Postmenstrual age ≥28 weeks	• Congenital anomalies • Generalized edema • Craniofacial abnormalities	NICU	April 2015-May 2016	2 neonatologists	• Specialized training in neonatal US • ICC 0.98 for interobserver reliability	Mid-sagittal view measuring ETT tip to aortic arch distance	Phased array (SonoSite M-Turbo)	8-4 MHz	• Aortic arch identification • ETT tip movement confirmation	Post-intubation (median 12 min)	Chest X-ray (T1-T2 vertebral level)	No	No
Takeuchi et al. (2019)	27.3 ± 2.1 weeks GA	Japan	Retrospective cohort	11 ELBW infants	• BW <1000g • Requiring intubation at birth	None specified	Delivery room/NICU	Jun 2016-Sep 2017	4 neonatologists	• 1 expert (4 yrs experience) • 3 trainees (1 yr experience) • No formal interpretation training	Neck ultrasound (thyroid level)	Linear array	13-6 MHz (body surface probe)	• Double trachea sign (esophageal) • Double line + comet-tail sign (tracheal)	Immediately post-intubation	Colorimetric CO2 detector + clinical confirmation	Yes	No
Vali et al. (2012)	30.2 ± 4.9 weeks GA	USA	Prospective pilot study	30 (31 ETTs)	Intubated neonates in NICU	Upper airway anomalies	NICU	Not specified	Neonatologist (experienced in US) & Respiratory therapist (trained for study)	Neonatologist with echocardiography expertise + trained respiratory therapist	Midsagittal view (upper sternum/neck) with ≤0.5cm ETT manipulation	Linear array	13	1. Hyperechoic ETT shadow 2. Right pulmonary artery as carina landmark 3. Dynamic ETT movement	Within 4 hours of CXR (mean 2.9±2.2 hours)	Chest radiograph (ETT tip-to-carina measurement)	No	Yes
Voulgaridou et al. (2025)	32.69 ± 4.42 weeks PMA	Greece	Prospective observational	67 (52 with guided US, 57 with independent US)	Intubated neonates <45 weeks postmenstrual age	Congenital anomalies, BW <500g, GA <23wks, generalized edema	NICU	Dec 2020-Sep 2022	NICU nurse	3 months theoretical + practical training (50 supervised scans)	ETT tip distance to RPA/aortic arch	GE Vivid T8: Curved array (8MHz) + Linear array (8-18MHz)	8-18 MHz	ETT tip position relative to vascular landmarks	Post-intubation	Chest X-ray	No	Yes
Zaytseva et al. (2018)	Gestational age: 31.6 ± 5.8 weeks	United States	Prospective observational study	75 (40 also underwent ultrasound assessment)	Neonates requiring oral intubation for: Cardiopulmonary failure, Respiratory distress, Surfactant administration	Major congenital anomalies	NICU (Neonatal Intensive Care Unit)	Not explicitly stated (published in 2018)	Neonatologist	Single operator (D.K.), neonatologist with extensive POC-US experience	High parasternal view; measured ETT–RPA distance using calipers	10 MHz cardiac probe (Zonare Z One PRO, Mindray, China)	10 MHz	ETT tip position relative to superior aspect of RPA (carina surrogate)	Immediately or shortly after CXR, same head position	Chest X-ray (CXR)	No	Yes

Supplementary Figure 1: Summary of risk of bias of included studies

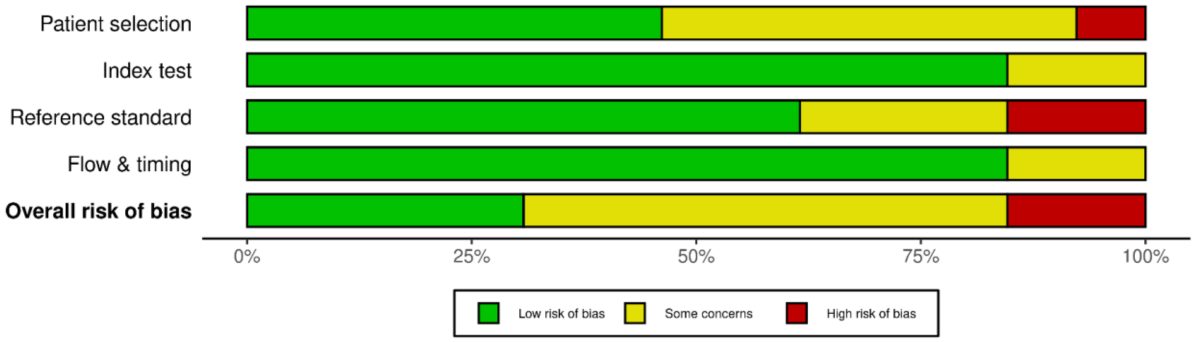

Supplementary Figure 2: Summary of applicability domains of included studies

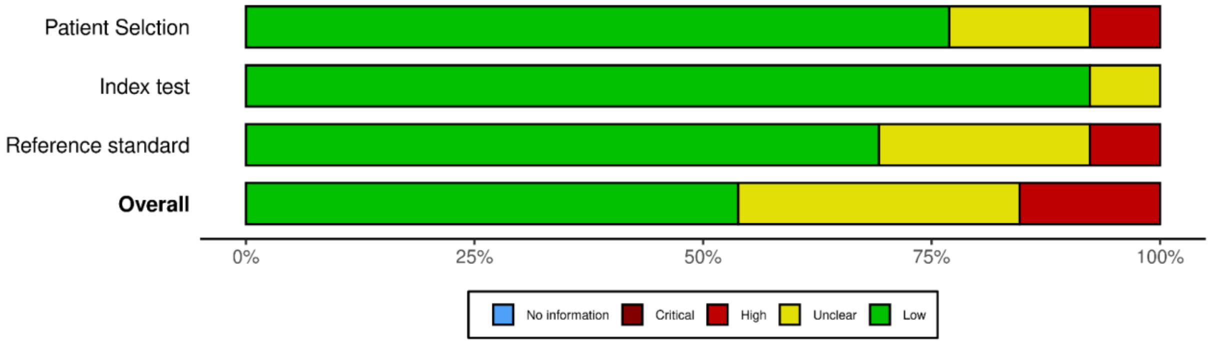

Supplementary Figure 3: Sensitivity analysis of POCUS-confirmed success rate

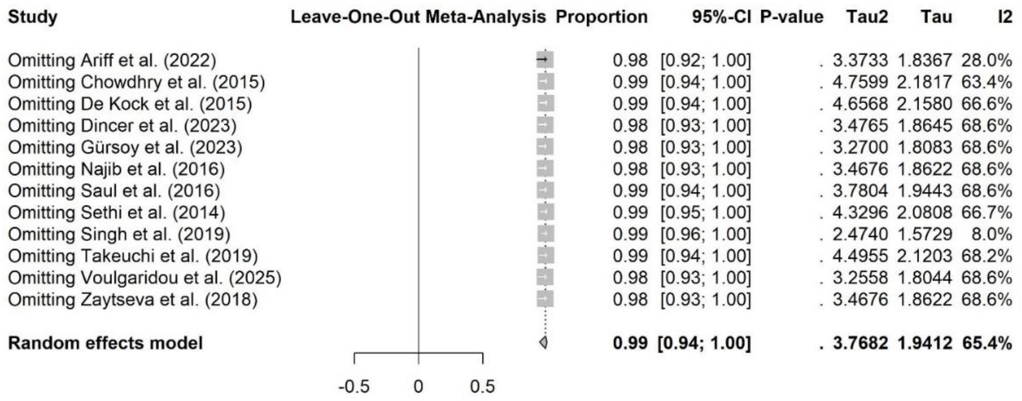

Supplementary Figure 4: Funnel plot of POCUS-confirmed success rate

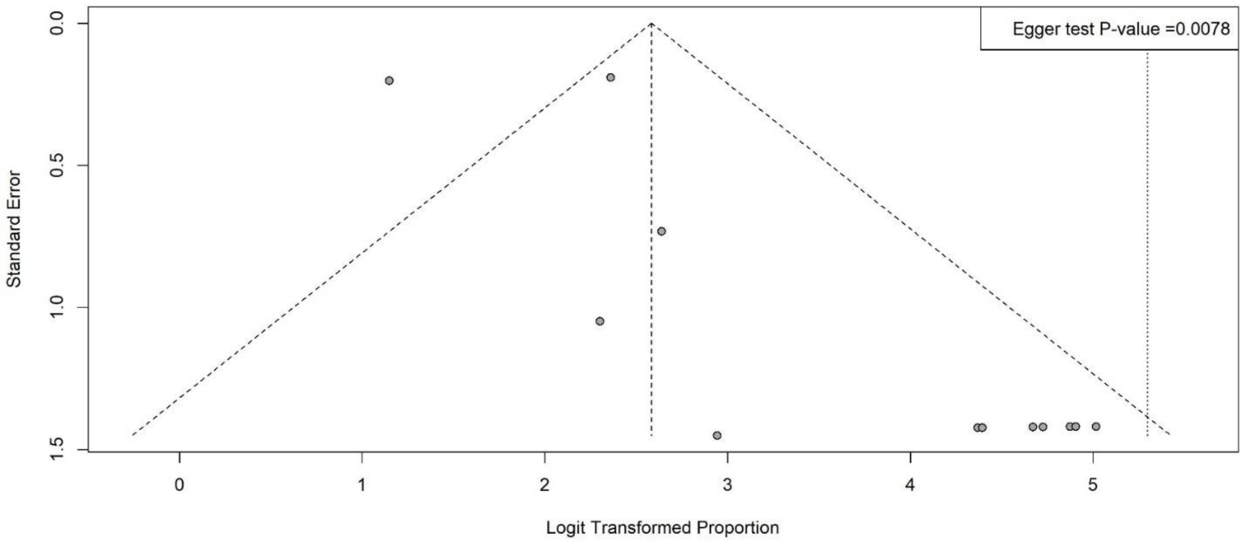

Supplementary Figure 5: Funnel plot of esophageal intubation

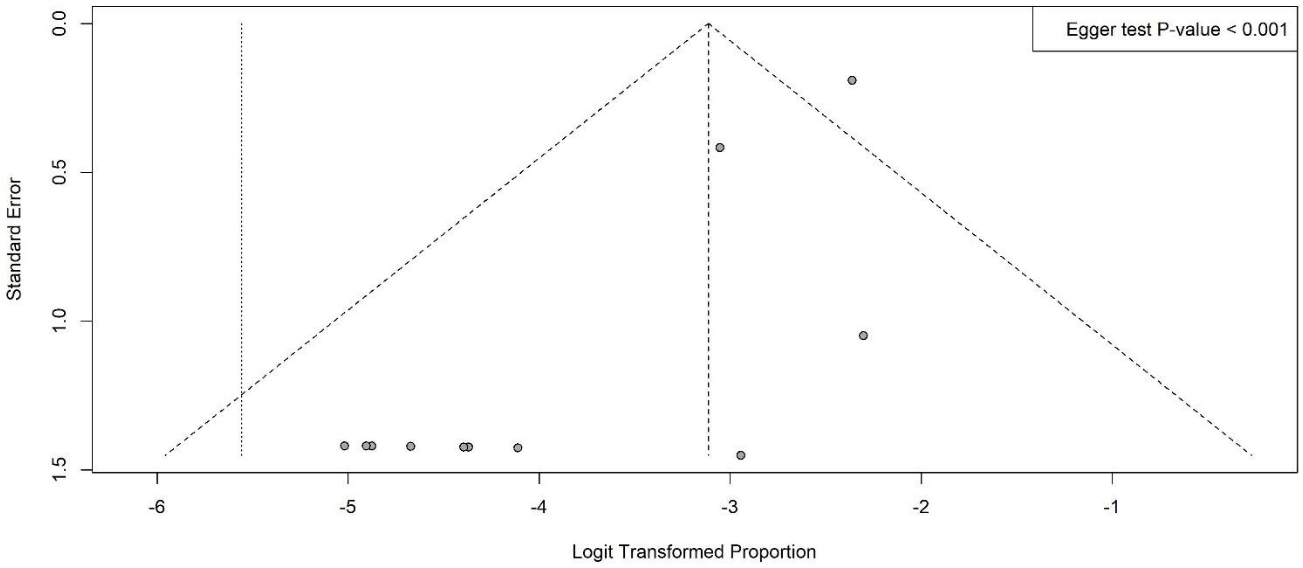

Supplementary Figure 6: Funnel plot of reintubation due to misplacement

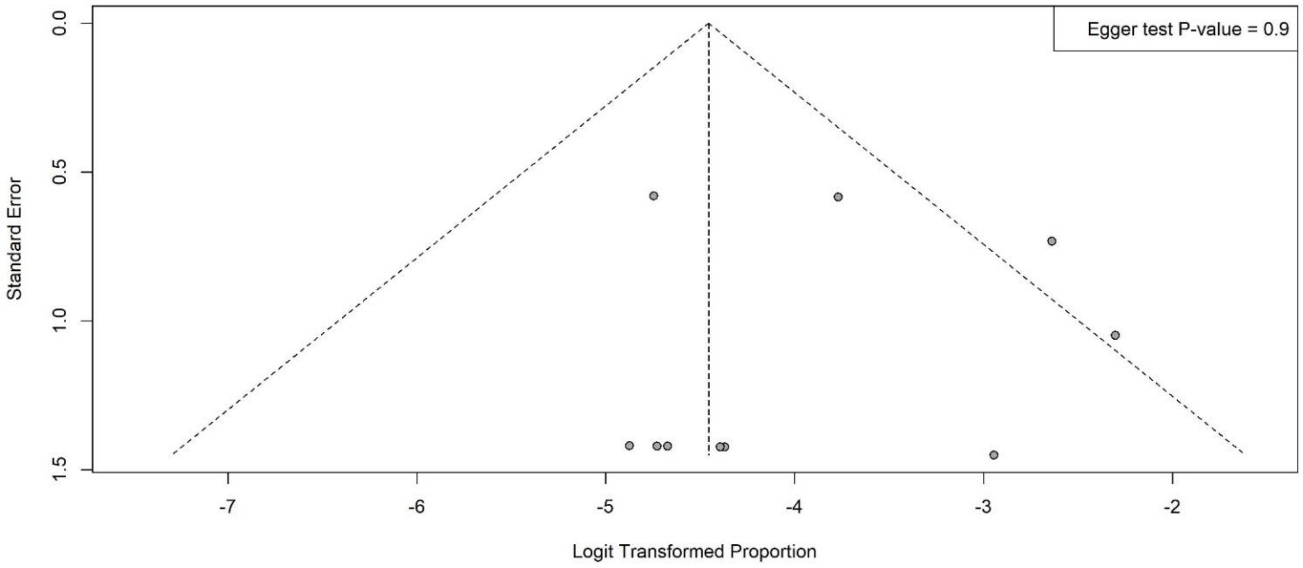

Supplement: Supplementary file 1 — Supplementary Information File [file 41372_2025_2461_MOESM1_ESM.pdf]
